# A rare case of mediastinal mass with massive pleural effusion misdiagnosed as neurogenic tumor

**DOI:** 10.1186/s13019-022-02082-4

**Published:** 2022-12-13

**Authors:** Wanyu Zheng, Xiaoyang Chen, Liyong Shi

**Affiliations:** 1 Nursing Department of the Second Affiliated Hospital of Fujian Medical University, Quanzhou, Fujian People’s Republic of China; 2 Department of Pulmonary and Critical Care Medicine, Respiratory Medicine Center of Fujian Province, The Second Affiliated Hospital of Fujian Medical University, 950 Donghai Street, Fengze District, Quanzhou, 362000 Fujian People’s Republic of China

**Keywords:** Mediastinum, Cavernous hemangiomas, Pleural effusion, Surgical resection

## Abstract

**Background:**

Mediastinal cavernous hemangiomas are extremely rare vascular tumors. To the best of our knowledge less than 20 cases of posterior mediastinal hemangioma have been reported in literature, and this is the first case of mediastinal cavernous hemangioma presenting with massive pleural effusion.

**Case presentation:**

We report a case of a 56-year-old female who presented with cough and chest tightness and was found with a massive pleural effusion in chest CT. It was mistaken for a malignant pleural effusion. A posterior mediastinal lesion was observed after thoracic drainage and misdiagnosed again as neurofibroma. The lesion was resected and post-operative histopathology suggested that it was a cavernous hemangioma. Post-operative recovery was uneventful, and a follow-up examination nearly 14 months later showed the patient had no recurrence.

**Conclusions:**

Due to the lack of diagnostic specificity and variety of clinical manifestations, CHM is often misdiagnosed prior to resection. This is the first description of mediastinal hemangioma presenting with massive pleural effusion. It is very important to consider mediastinal hemangioma before operation to reduce surgical complications, and it should be in the differential diagnosis of posterior mediastinal masses.

## Background

Mediastinal cavernous hemangioma (CHM) is a rare benign tumor with an incidence of less than 0.5% of mediastinal masses [1]. Most of CHMs are located in the anterior mediastinum, whereas the posterior mediastinum is rarely involved [2]. Mediastinal hemangioma presenting with massive pleural effusion has not been reported before. It is difficult to diagnose preoperatively. Treatment options include sclerotherapy, embolization, and surgical resection. Complete resection of CHM has a good prognosis [3]. We report a case of 56-year-old female present with massive pleural effusion, which demonstrated posterior mediastinal cavernous hemangioma on resection, with no recurrence thus far.

## Case presentation

**Fig. 1:**
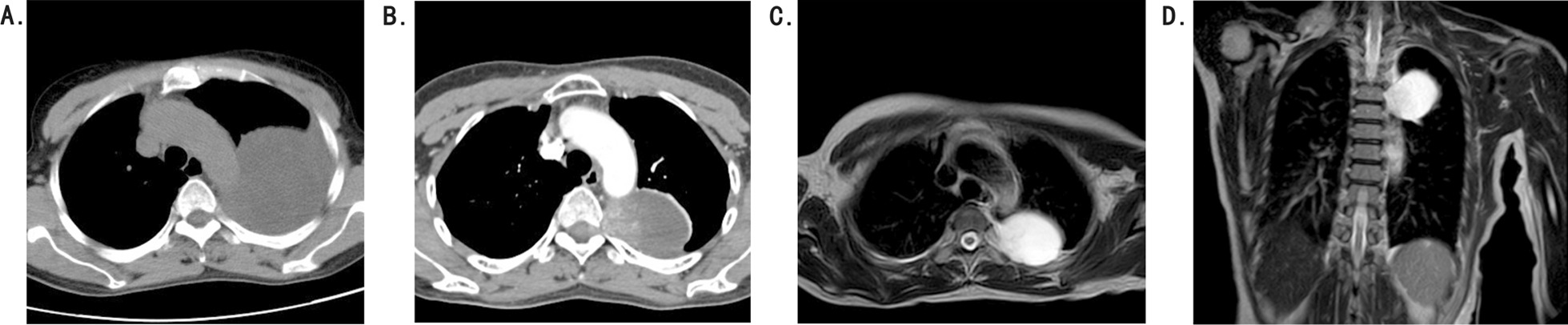
CT showing a large left-side pleural effusion (**A**). Enhanced CT showing a posterior mediastinal mass (**B**). T2-weighted contrast-enhanced sections showing heterogeneously enhanced (**C**), Axial and (**D**), coronal images

A 56-year-old nonsmoking female presented with cough and chest tightness for 20 days with no past medical history. Vital signs were stable except diminished left lung sounds were noted in chest auscultation. She was then found to have a large left-sided pleural effusion in CT scan (Fig. 1A). Pleurocentisis revealed a clear fluid with yellow supernatant. The laboratory analysis of the fluid revealed the following: total protein, 41 g/L (serum protein, 67 g/L) and WBC count 3700 cells/ml (13% polymorphs and 87% lymphocytes). No organisms were seen on Gram stain, and no acid-fast bacilli ware seen on auramine stain. Cultures of the pleural effusion specimens and tumor markers were also negative. No malignant cells were seen, only mesothelial cells and lymphocytes. An enhanced CT scan performed one week later indicated a 6.2 cm × 4 cm oval-shaped lesion with significant enhancement, with maximum enhanced CT value approximately 125Hu (Fig. 1B). Magnetic resonance imaging (MRI) demonstrated a 6.2 cm × 4 cm well-defined, oval-shaped lesion with suspected invasion of intervertebral foramen, placed in the left costovertebral space (Fig. 1C, D). Before operation, it was diagnosed as neurogenic tumor.

Complete resection was performed by video-assisted thoracoscopic surgery (VATS). On direct visualization, there was a lesion in the posterior mediastinum close to left paravertebral region with moderate hardness, not adhering to the surrounding spine, mediastinum or chest. The capsule was complete and the boundary was clear. Thoracoscopic resection was performed successfully, and the estimated intraoperative bleeding was 200 ml. The size of lesion was approximately 6 cm × 5 cm × 3.5 cm and was sent for routine pathology.

**Fig. 2:**
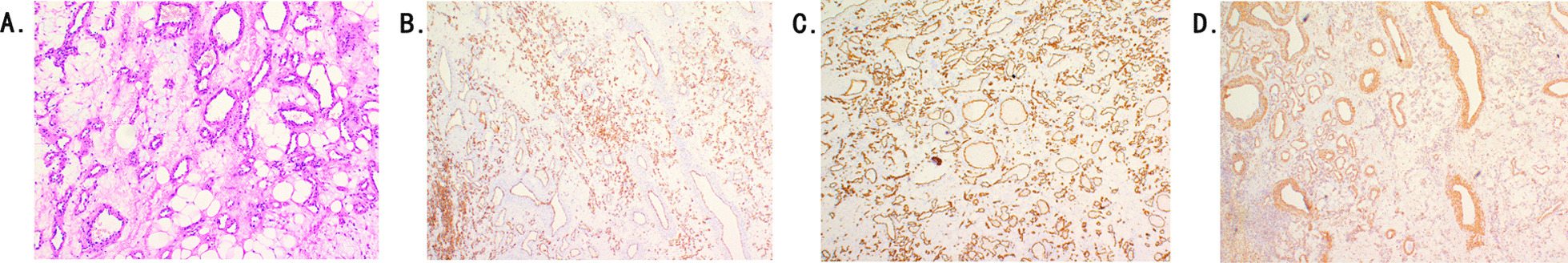
Magnification showing vascular lacunas with flattened endothelium,100 × (**A**). Immunostaining was positive for CD 31 (**B**), CD34 (**C**) and SMA (**D**)

Histopathology revealed a cavernous hemangioma composed of varying size and dilated cavernous sinuses, with an inner wall lined with vascular endothelium (Fig. 2A). In addition, makers including CD34, CD31 and SMA positive (Fig. 2C, D), Ki-67 staining was positive (2%). The patient’s post-operative recovery was uneventful and discharged from hospital on the third day after surgery. No recurrence was not noted at 14 month follow-up.

## Discussion

Mediastinal cavernous hemangioma (CHM) is a rare disease of mediastinal benign tumor originating from vascular endothelial cells which is caused by abnormal vascular development during embryonic period. The disease mostly occurs in young adults, and the gender difference is not large [2]. It tends to present at various sites, sometimes combined with hemangioma lesions in other parts of the skin, liver, spleen, and kidney [4]. To the best of our knowledge less than 20 cases of posterior mediastinal hemangioma have been reported in literature in English.

Preoperative diagnosis of CHM is particularly difficult. All the cases that have been reported so far were diagnosed after surgical resection. Although most cases demonstrated heterogeneous well-circumscribed masses, punctate, and rounded calcifications, some display a wide range of radiological features like dumbbell tumors or infiltrative appearance [5]. On CT scans, mediastinal schwannomas appear as well-defined, homogeneous masses showing heterogenous mild to moderate enhancement following intravenous contrast administration. The MR features of ancient schwannomas include similar signal characteristics to skeletal muscle on T1-weighted sequences. Hyperintense areas, which appear on T2-weighted images, correspond to cystic degeneration in the tumor [6]. Multiphase enhanced CT and MRI scan are valuable in the diagnosis of hemangioma, where the tumor shows peripheral nodular enhancement on early phase images and progressive centripetal fill-in on delayed phase images when a “fast in and slow out” protocol was performed [7].

We illustrated a case of CHM in the posterior with massive pleural effusion, which is an extremely rare condition. The clinical manifestations are non-specific and can be easily misdiagnosed as schwannoma. The etiology of the pleural effusion is unclear. We speculate that repeated friction of the base of the focus close to the vertebrae leads to fistula and aseptic pleurisy. However, during the operation, the capsule of focus is complete and no visible fistula except the very thin top of the tumor. This is just speculation and real mechanism is still unknown.

Misdiagnosis can lead to percutaneous lung biopsy, inappropriate surgical planning and serious complications, especially life-threatening hemorrhage. If hemangioma can be considered before operation, preoperative interventional embolization is a method recently recommended to control intraoperative bleeding and even treat hemangioma [8], and is the preferred plan of hemostasis. Complete resection of CHM has a good prognosis, and the disease does not commonly recur. Thoracoscopy is the mainstay of treatment. When the lesion is difficult to remove, an open thoracotomy should be considered early to avoid operative complication [8].

## Conclusion

Mediastinal hemangioma presenting with massive pleural effusion may be the first to describe to the best of our knowledge. The patient was performed thoracoscopic surgical resection successfully, which demonstrated the diagnosis of posterior mediastinal cavernous hemangioma with no recurrence till now.

## Data Availability

All data and materials are available upon reasonable request from the corresponding author.

## Abbreviations

CHM: Mediastinal cavernous hemangioma
MRI: Magnetic resonance imaging
VATS: Video-assisted thoracoscopic surgery
